# Association between healthy eating index-2015 and abdominal aortic calcification among US Adults

**DOI:** 10.3389/fnut.2022.1027136

**Published:** 2023-01-18

**Authors:** Jundi Jia, Jie Zhang, Dan Ma, Zihao Zhang, Lin Zhao, Tongxin Wang, Hao Xu

**Affiliations:** ^1^Graduate School, Beijing University of Chinese Medicine, Beijing, China; ^2^National Clinical Research Center for Chinese Medicine Cardiology, Xiyuan Hospital, China Academy of Chinese Medical Sciences, Beijing, China; ^3^Graduate School, China Academy of Chinese Medical Sciences, Beijing, China

**Keywords:** healthy eating index, abdominal aortic calcification, vascular calcification, cardiovascular disease, NHANES

## Abstract

**Aims:**

To evaluate the relationship of the healthy eating index-2015 (HEI-2015) with abdominal aortic calcification (AAC) in US adults.

**Methods:**

We conducted a cross-sectional study with data extracted from the National Health and Nutrition Examination Survey (NHANES). AAC score was measured using the scoring system of Kauppila (AAC-24) and Schousboe (AAC-8). HEI-2015, which was used for evaluating compliance with Dietary Guidelines for Americans (DGA), was calculated through two rounds of 24-h recall interviews. HEI-2015 was categorized as inadequate (<50), average (50~70), and optimal (≥70) groups for analysis, while the AAC-24 score was grouped by whether the score was >0. Weighted multiple regression analyses were conducted to estimate the association of HEI-2015 with AAC score and the presence of AAC. Moreover, smooth curve fittings, based on a generalized additive model (GAM), were applied to evaluate a possible non-linear relationship. Sensitivity analysis and subgroup analysis were performed to provide more supporting information.

**Results:**

A total of 2,704 participants were included in the study (mean age, 57.61 ± 11.40 years; 51.78% were women). The mean score of HEI-2015 was 56.09 ± 13.40 (41.33 ± 6.28, 59.44 ± 5.54, and 76.90 ± 5.37 for inadequate, average, and optimal groups, respectively). After adjusting for covariates, higher HEI-2015 was associated with decreased AAC score (AAC-24: β = −0.121, 95% CI: −0.214, −0.028, *P* = 0.010; AAC-8: β= −0.054, 95% CI: −0.088, −0.019, *P* = 0.003) and lower risk of AAC (OR = 0.921, 95% CI: 0.855, 0.993, *P* = 0.031). Among the components of HEI-2015, a higher intake of fruits, greens, and beans was associated with a lower AAC score. Subgroup analysis showed that an inverse association of HEI-2015 with AAC score existed among different groups.

**Conclusion:**

The study presented that higher HEI-2015 was related to a lower AAC score and decreased risk of having AAC, indicating that greater compliance with 2015–2020 DGA, assessed by HEI-2015, might be beneficial for preventing vascular calcification and CVD among US adults.

## 1. Introduction

Cardiovascular disease (CVD) is the prominent cause of mortality and disability both in the United States (US) and around the world, leading to a heavy burden on the medical system (1). CVD prevention is a matter of concern for research and health policy (2). Vascular calcification, featured with the pathological deposits of calcium phosphate crystals in the arterial intima (3), is a potential risk factor for the progression and rupture of atherosclerotic plaques and has been highly associated with CVD events (4). It is known that in diabetes and chronic kidney disease (CKD), vascular calcification progresses more rapidly, suggesting a higher risk of cardiovascular events in the future (5, 6). The mechanism of vascular calcification is quite complicated and not well-defined, possibly related to uninhibited mineralization, homeostasis of calcium and phosphorus, osseous differentiation of vascular cells, paracrine factors, cell death, and substrate degradation (7). Furthermore, the current treatments for calcification are limited. Although calcium channel blockers (CCBs) were observed to have rewarding effects on vascular calcification in animal studies, their effects in humans were minimal (8, 9). Sodium thiosulfate was found to have significant effects on vascular calcification in rats (10), but its clinical efficacy and safety have not been established. Pyrophosphate can be used as a potential treatment due to the loss of extracellular pyrophosphate synthesis which likely leads to vascular calcification, but its use is limited by pharmacokinetics and stability (11). Thus, there are no treatments with sufficient evidence to reverse vascular calcification or prevent it at an early stage. It is known that the presence and extent of coronary artery calcification are associated with coronary artery events, independent of other CVD risk factors (12). Therefore, abdominal aortic calcification (AAC), which happens earlier than coronary artery calcification, is a latent indicator associated with vascular morbidity and mortality and can be used to predict subclinical CVD (13, 14). A retrospective study demonstrated that AAC was a sensitive predictive factor of cardiovascular events, superior to the Framingham risk score in asymptomatic adults (15). The measuring method of AAC uses the marking system of Kauppila (16) and Schousboe (17), which has been generally applied.

Dietary factors play a crucial role in the primary prevention of CVD and have attracted much attention due to their modifiability (18). At present, many factors such as dietary folate intake, polyphenols, and eicosapentaenoic acid, have been found to be possibly beneficial to the prevention of CVD (19–21). However, food and nutrients are often consumed in various combinations. Dietary patterns formed by the combination of foods and nutrients can better reflect the real-world dietary condition by taking into account the latent interactions and cumulative correlations between different dietary components (22, 23). Several studies have focused on the relationship between dietary patterns and CVD. A population-based cohort study in British showed that the Dietary Approaches to Stop Hypertension (DASH) dietary pattern was inversely associated with incident CVD and CVD mortality (24). A study of three prospective cohorts with 32 years of follow-up demonstrated that greater adherence to the HEI-2015 was correlated with a lower risk of CVD among US adults (25). Furthermore, we need to understand the relationship between dietary patterns and vascular calcification in order to provide some evidence for preventing vascular calcification and CVD from a macroscopic perspective of diet. HEI-2015 was used for evaluating compliance with the Dietary Guidelines for Americans (DGA) updated every 5 years and was selected to assess its association with vascular calcification in our study. Moreover, since the abdominal aorta is more prone to calcify (26), studies on AAC are more conducive to early prevention. To the best of our knowledge, there is no research examining the association of HEI-2015 with AAC. Therefore, we conducted a cross-sectional study to investigate whether higher HEI-2015 is linked to lower AAC scores and reduced risk of AAC in US adults.

## 2. Methods

### 2.1. Study population

The NHANES, applying a stratified, multistage probability sample of non-institutionalized civilians, is a cross-sectional investigation to obtain basic information and healthy condition. National Center for Health Statistic (NCHS) is responsible for the implementation of NHANES. NCHS Ethics Review Board had approved the protocols and subjects had signed the informed consent (27).

**Figure 1:**
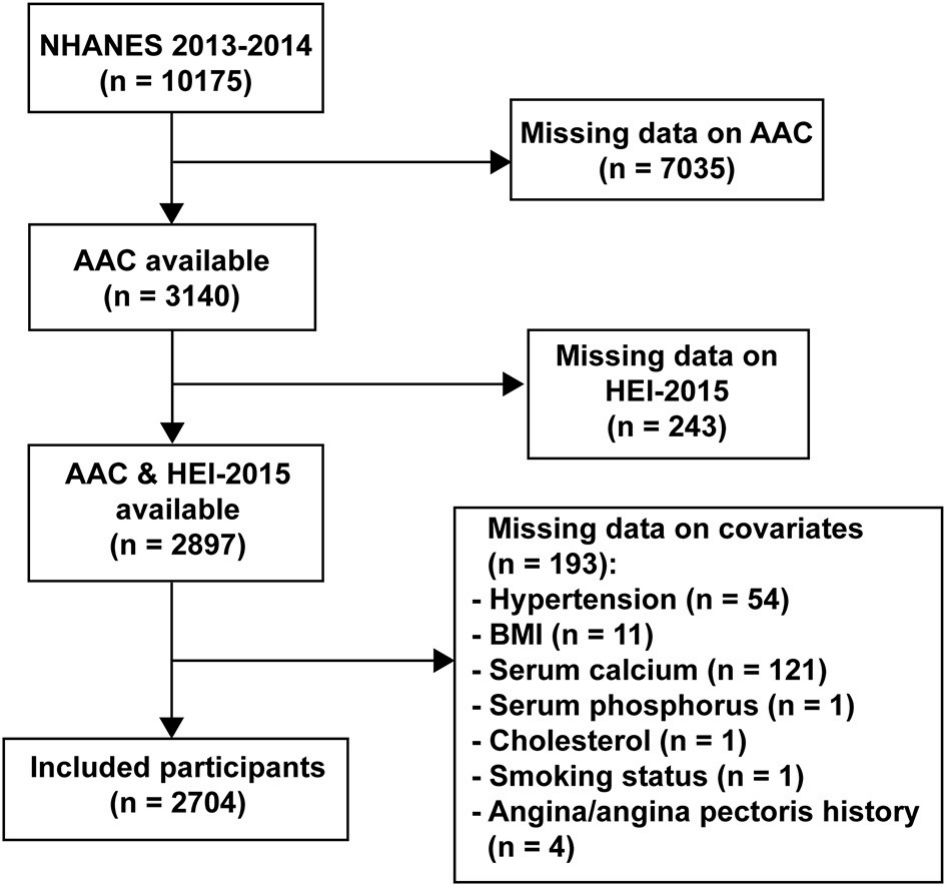
The flowchart.

Data were chosen from the 2013–2014 NHANES cycle, which is the only cycle that included the information on AAC. Due to the usage of dual-energy X-ray absorptiometry (DXA) for the measure of calcification, subjects who were pregnant, <40 years old, and had self-reported radiation exposure within the past 7 days or of >450 pounds were not allowed to participate. Subjects with data on both HEI-2015 and AAC scores were enrolled in this study (*n* = 2,897). After excluding subjects with missing data on covariates, 2,704 participants were eligible for the analysis (Figure 1).

### 2.2. Study variables

HEI-2015 was the independent variable of the study. Dietary data of NHANES included the types and amounts of food and beverages consumed within 24 h before the interview, with the further calculation of energy, nutrients, and other food components. Food Surveys Research Group (FSRG) for agriculture in the US was in charge of dietary data collection, database maintenance, and data auditing. Data from the dietary interview of the first recall were personally collected at the Mobile Examination Center (MEC) and the second interview was collected by phone after 3–10 days. United States Department of Agriculture (USDA) Food Patterns Equivalence Database was used for translating dietary data into standardized quantities of food groups to calculate HEI-2015. HEI-2015 consists of 13 dietary components, nine of which are adequacy components including whole grains, dairy, total protein foods, total fruits, whole fruits, greens and beans, total vegetables, seafood and plant proteins, and fatty acids, and the rest are moderation components including refined grains, sodium, added sugars, and saturated fats (28). The scoring is based on an energy density of 1,000 calories, except for fatty acids, which is a ratio of unsaturated to saturated fatty acids (29). A higher intake of adequacy components or a lower intake of moderation components results in a higher HEI-2015 score, meaning greater compliance with the 2015–2020 DGA recommendations. The highest intake scored 10 points in three adequacy components (whole grains, dairy, and fatty acids) and 0 points in all moderation components, while the lowest intake scored 0 and 10 points, respectively. The other adequacy components scored 5 points in the highest intake and 0 points in the lowest intake. HEI-2015 is the sum of the component scores, ranging from 0 to 100 points. We categorized HEI-2015 as inadequate (<50), average (50~70), and optimal (≥70) group for analysis, according to the previous study (30).

The AAC score was extracted from the examination data of NHANES. Lateral spine images, acquired using DXA, could allow precise identification of AAC. A DXA scan reduces radiant exposure, and its image resolution of lateral spine scans is comparable to those seen with standard X-rays. Instant Vertebral Assessment (IVA) lateral spine scans, which were obtained on the Hologic Discovery model. A densitometer was used for AAC measurement. For each participant's scan result, the UCSF was responsible for the review and analysis to ensure that the scan analysis was accurate. Using Kauppila's scoring approach, the anterior and posterior walls of the aorta were split up into four parts, which were in close proximity to the L1–L4 lumbar vertebrae. The four parts are scored from 0 to 6 according to the degree of calcification, with a total score range of 0 to 24. According to previous studies, the Kauppila score of >0 indicated the presence of AAC (31). Additionally, we conducted the sensitivity analysis using the AAC-8 score (Schousboe score), with a total score range of 0–8. AAC-8 correlated with AAC-24 (Kauppila score) has the advantage that it is less influenced by specked calcification dispersed in eight segments, although it requires more skills (32).

According to the clinical relevance, demographics [gender, age, race/ethnicity, and body mass index (BMI)], metabolic indicators (cholesterol, triglycerides, creatinine, serum calcium, and serum phosphorus), comorbidities (hypertension, diabetes, and angina/angina pectoris history), lifestyles (smoking status, alcohol consumption, and daily energy intake), and socioeconomic features (education and poverty ratio) were included as potential covariates. The data on demographics can be found in the demographics section of NHANES, meanwhile, the information on metabolic indicators and lifestyles can be obtained from laboratory data and questionnaire data, respectively. Detailed information on the covariates was presented in Supplementary Table 1, and data and description of all the above variables are available for free at www.cdc.gov/nchs/nhanes/.

### 2.3. Statistical analyses

Weighted analysis was used as recommended by the NCHS, considering the oversampling of minorities to provide an accurate estimate of effects for the population (33) because of the complex multi-stage cluster survey design of NHANES. Continuous variables were shown as population-weighted means with standard error (SE), while categorical variables were presented as frequency (percentage). The HEI-2015 was categorized into three groups for analysis. Population-weighted chi-square test (categorical variables) and population-weighted linear regression model (continuous variables) were used for calculating the difference of the baseline characteristics. Weighted multiple regression analyses, taking into account both population weights and dietary weights, were conducted to explore the independent association of HEI-2015 with AAC. Then, stratified multivariate regression was used for subgroup analysis. Meanwhile, the smooth curve fittings, based on GAM, were conducted to observe the linear or non-linear relationship of HEI-2015 with AAC. A *P*-value of <0.05 was considered significant. R version 3.4.3 (http://www.R-project.org, The R Foundation) and Empower software (www.empowerstats.com; X&Y Solutions, Inc., Boston MA) were used for statistical analysis.

## 3. Results

### 3.1. Baseline

The weighted demographic features of the 2,704 subjects in the study grouped by HEI-2015 score are shown in Table 1. The mean age of the subjects was 57.61 ± 11.40 years. Of these subjects, 51.78 % were women and 70.80 % were non-Hispanic white. The average score of HEI-2015 was 56.09 ± 13.40 (41.33 ± 6.28, 59.44 ± 5.54, and 76.90 ± 5.37 for inadequate, average, and optimal groups, respectively). The subjects in the optimal group of HEI-2015 were more likely to be older, women, white, non-smokers, not impoverished, have higher education level, lower BMI, less alcohol intake and daily energy intake, lower level of creatinine and triglycerides, and a higher level of serum calcium and serum phosphorus.

**Table 1:** Characteristic of participants in the study, weighted.

**Variables**	**Groups of HEI-2015**			**All: 56.09 ± 13.40 (*n =* 2704)**	***P*** **-value**
	Inadequate^*^: 41.33 ±**6.28 (*****n** =* **935, 34.58%)**	Average^*^: 59.44 ±**5.54 (*****n** =* **1325, 49.00%)**	Optimal^*^: 76.90 ±**5.37 (*****n** =* **444, 16.42%)**		
Age (years)	55.65 ± 10.99	58.28 ± 11.59	59.78± 11.03	57.61 ± 11.40	<0.0001
**Gender**, ***N*** **(%)**					
Male	484 (54.51)	643 (47.32)	176 (37.31)	1303 (48.22)	
Female	451 (45.49)	682 (52.68)	268 (62.69)	1401 (51.78)	<0.0001
**Race/ethnicity**, ***N*** **(%)**					
Hispanic	194 (11.56)	321 (11.93)	101 (10.81)	616 (11.62)	
Non-hispanic white	461 (70.03)	590 (71.48)	180 (70.29)	1231 (70.80)	
Non-hispanic black	199 (12.18)	242 (8.89)	70 (7.52)	511 (9.81)	
Others	81(6.23)	172 (7.70)	93 (11.38)	346 (7.77)	0.0037
**Education**, ***N*** **(%)**					
High school or below	506 (47.30)	555 (34.08)	134 (22.45)	1195 (36.79)	
Above high school	429 (52.70)	770 (65.92)	310 (77.55)	1509 (63.21)	<0.0001
**Poverty ratio**, ***N*** **(%)**					
≤ 1.3	351 (25.87)	352 (17.50)	90 (11.47)	793 (19.48)	
>1.3	584 (74.13)	973 (82.50)	354(88.53)	1911 (80.52)	<0.0001
BMI (kg/m^2^)	29.13± 5.61	28.34 ± 5.27	27.29 ± 5.68	28.45 ± 5.49	<0.0001
Cholesterol (mg/dL)	198.59 ± 45.38	196.99 ± 40.64	196.44± 42.15	197.45 ± 42.57	0.5879
Triglycerides (mg/dL)	177.20 ± 201.36	159.69 ± 108.40	139.47 ± 105.33	162.53 ± 147.33	<0.0001
Creatinine (mg/dL)	0.94 ± 0.36	0.92 ± 0.42	0.87 ± 0.25	0.92 ± 0.38	0.0054
Serum phosphorus (mg/dL)	3.76 ± 0.56	3.83 ± 0.58	3.90 ± 0.52	3.81 ± 0.56	<0.0001
Serum calcium (mg/dL)	9.43 ± 0.36	9.47 ± 0.37	9.48 ± 0.32	9.46 ± 0.36	0.0026
**Hypertension**, ***N*** **(%)**					
Yes	322 (32.07)	449 (29.24)	156 (33.87)	927 (30.94)	
No	613 (67.93)	876 (70.76)	288 (66.13)	1777 (69.06)	0.1297
**Diabetes**, ***N*** **(%)**					
Yes	201 (17.85)	323 (19.01)	87 (15.32)	611 (18.03)	
No	734 (82.15)	1002 (80.99)	357 (84.68)	2093 (81.97)	0.2228
**Angina/Angina pectoris history**, ***N*** **(%)**					
Yes	32 (2.68)	43 (3.22)	11 (2.79)	86 (2.97)	
No	903 (97.32)	1282 (96.78)	433 (97.21)	2618 (97.03)	0.7431
**Smoking status**, ***N*** **(%)**					
Never smoking	409 (43.63)	749 (58.11)	304 (64.75)	1462 (54.18)	
Former smoker	265 (26.66)	194 (12.58)	28 (8.83)	487 (16.83)	
Current smoker	261 (29.71)	382 (29.31)	112 (26.42)	755 (28.99)	<0.0001
Alcohol consumption (drinks/*N* %)	210.00± 319.94	258.20 ± 387.21	201.19 ± 236.94	232.71± 345.77	0.0006
Current drinkers	701 (80.69)	958 (78.31)	297 (76.48)	1956 (78.83)	
Ex-drinkers	122 (10.59)	163 (9.94)	77 (11.46)	362 (10.40)	
Non-drinkers	112 (8.72)	204 (11.75)	70 (12.06)	386 (10.77)	0.1478
Daily energy intake (kcal/d)	4060.60 ± 1765.88	3839.82 ±1541.73	3604.79 ±1246.44	3878.77 ± 1589.43	<0.0001

Percentage for Categorical variables, mean ± SE for continuous variables. SE, standard error of the estimate. BMI, body mass index; HEI, healthy eating index. A drink: a 12-oz. beer, a 5-oz. glass of wine, or one and a half ounces of liquor. ^*^Inadequate: HEI-2015 score < 50; Average: 50 ≤ HEI-2015 score < 70; Optima: HEI-2015 score ≥ 70.

### 3.2. Inverse relationship of HEI-2015 with AAC score and the presence of AAC

Multiple regression models were applied to analyze the relationship between HEI-2015 and AAC in different models. Model 1 was adjusted for gender, BMI, race/ethnicity, and age. We added indicators of cholesterol, triglycerides, creatinine, serum calcium, and serum phosphorus for the adjustment in Model 2 and adjusted for all covariates in Model 3. We calculated β-coefficients and odds ratio of the AAC per 10-unit increments of HEI-2015. In Table 2, an inverse relationship of HEI-2015 with AAC-24 score was observed in all models (Model 3: β = −0.121, 95% CI: −0.214, −0.028, *P* = 0.010). When the HEI-2015 was used as a categorical variable and the inadequate group was used as a reference one, the HEI-2015 in the optimal group was associated with lower AAC score in all models, implying that optimal intake was beneficial for reducing AAC score (Model 3: β = −0.471, 95% CI: −0.843, −0.099, *P* = 0.013, P for trend = 0.02). When the AAC was shown as a categorical variable, this inverse trend was found in all models. Higher HEI-2015 was related to a lower risk of having AAC (Model 3: OR = 0.921, 95% CI: 0.855, 0.993, *P* = 0.031). This meant that for every 10-unit increment of HEI-2015, the risk of having AAC was roughly 7.9% lower. Consistent results were found between HEI-2015 and AAC-8 in all models (Model 3: β = −0.054, 95% CI: −0.088, −0.019, *P* = 0.003). In addition, compared with the inadequate group, the optimal group of HEI-2015 had a lower AAC-8 score with statistical significance (Model 3: β = −0.189, 95% CI: −0.327, −0.050, *P* = 0.008, P for trend = 0.01).

**Table 2:** Associations of 10-unit increments of HEI-2015 with AAC score and the risk of AAC.

	**Model 1**		**Model 2**		**Model 3**	
	β**/OR (95% CI)**	***P*** **-value**	β**/OR (95% CI)**	***P-*** **value**	β**/OR (95% CI)**	***P-*** **value**
Risk of AAC	0.880 (0.822, 0.942)	<0.001	0.882 (0.824, 0.945)	<0.001	0.921 (0.855, 0.993)	0.031
AAC-24 score	−0.188 (−0.275, −0.102)	<0.001	−0.179 (−0.266, −0.093)	<0.001	−0.121 (−0.214, −0.028)	0.010
**Groups of HEI-2015**						
Inadequate	Reference		Reference		Reference	
Average	−0.202 (−0.454, 0.051)	0.117	−0.193 (−0.445, 0.060)	0.135	−0.093 (−0.361, 0.176)	0.499
Optimal	−0.693 (−1.044, −0.342)	<0.001	−0.653 (−1.004, −0.301)	<0.001	−0.471 (−0.843, −0.099)	0.013
P for trend	<0.001		<0.001		0.02	
AAC-8 score	−0.079 (−0.111, −0.047)	<0.001	−0.076 (−0.108, −0.043)	<0.001	−0.054 (−0.088, −0.019)	0.003
**Groups of HEI-2015**						
Inadequate	Reference		Reference		Reference	
Average	−0.099 (−0.193, −0.005)	0.040	−0.094 (−0.188, 0.001)	0.051	−0.052 (−0.152, 0.048)	0.304
Optimal	−0.274 (−0.404, −0.143)	<0.001	−0.258 (−0.389, −0.127)	<0.001	−0.189 (−0.327, −0.050)	0.008
P for trend	<0.001		<0.001		0.01	

Model 1: Age, gender, race, and BMI were adjusted; Model 2: Model 1 + cholesterol, triglycerides, creatinine, serum phosphorus, and serum calcium; Model 3: Model 2 + hypertension, diabetes, angina/angina pectoris history, smoking status, alcohol consumption, daily energy intake, education, and poverty ratio. HEI-2015, Healthy eating index-2015. CI, confidence intervals; OR, odds ratio.

In an analysis exploring the relationship between components of HEI-2015 and AAC score (Table 3), combining the results of AAC-24 with AAC-8, higher intake of greens and beans (AAC-24: β= −0.093, 95% CI: −0.148, −0.038, P < 0.001; AAC-8: β= −0.031, 95% CI: −0.051, −0.010, *P* = 0.004), total fruits (AAC-24: β= −0.096, 95% CI: −0.160, −0.032, *P* = 0.003; AAC-8: β= −0.042, 95% CI: −0.066, −0.019, *P* < 0.001), and whole fruits (AAC-24: β= −0.069, 95% CI: −0.127, −0.011, *P* = 0.020; AAC-8: β= −0.031, 95% CI: −0.052, −0.009, *P* = 0.005) was associated with a statistically significant reduction in AAC score, after adjusting for all covariates.

**Table 3:** Associations between components of HEI-2015 and AAC score.

	**AAC-24**		**AAC-8**	
	β **(95%CI)**	***P-*** **value**	β **(95%CI)**	***P*** **-value**
**Components of HEI-2015**				
Total vegetables	0.009 (−0.073, 0.092)	0.826	−0.001 (−0.032, 0.030)	0.949
Greens and Beans	−0.093 (−0.148, −0.038)	<0.001	−0.031 (−0.051, −0.010)	0.004
Total fruits	−0.096 (−0.160, −0.032)	0.003	−0.042 (−0.066, −0.019)	<0.001
Whole fruits	−0.069 (−0.127, −0.011)	0.020	−0.031 (−0.052, −0.009)	0.005
Whole grains	−0.033 (−0.069, 0.003)	0.071	−0.007 (−0.020, 0.007)	0.320
Total dairy	0.015 (−0.025, 0.055)	0.460	0.003 (−0.019, 0.018)	0.684
Total protein foods	−0.039 (−0.165, 0.086)	0.541	−0.019 (−0.066, 0.028)	0.434
Seafood and plant proteins	−0.013 (−0.070, 0.044)	0.657	−0.013 (−0.034, 0.008)	0.234
Fatty acids	−0.018 (−0.053, 0.017)	0.317	−0.007 (−0.020, 0.006)	0.287
Refined grains	0.023 (−0.013, 0.060)	0.209	0.004 (−0.010, 0.017)	0.610
Sodium	−0.029 (−0.066, 0.009)	0.139	−0.012 (−0.026, 0.003)	0.111
Added sugars	−0.017 (−0.058, 0.024)	0.411	−0.009 (−0.024, 0.007)	0.262
Saturated fats	−0.025 (−0.063, 0.013)	0.203	−0.012 (−0.026, 0.002)	0.101

Adjusted for age, gender, race, BMI, cholesterol, triglycerides, creatinine, serum phosphorus, serum calcium, hypertension, diabetes, angina/angina pectoris history, smoking status, alcohol consumption, daily energy intake, education, and poverty ratio. CI, confidence intervals. HEI-2015, Healthy eating index-2015.

**Figure 2:**
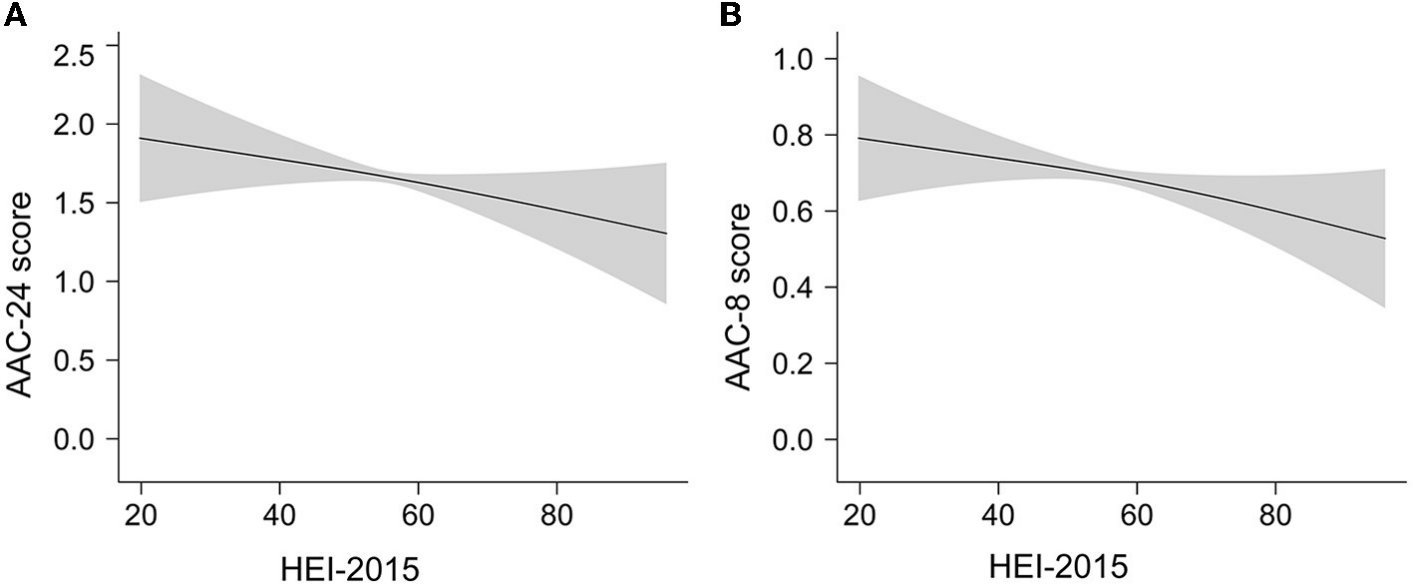
The linear dose–response relationship between HEI-2015 and AAC score by GAM. The shaded areas represent 95% confidence intervals. **(A)** The linear relationship of HEI-2015 with AAC-24 score. **(B)** The linear relationship of HEI-2015 with AAC-8 score.

Smooth curve fittings based on GAM were conducted to evaluate a possible non-linear dose–response relationship of HEI-2015 with AAC. The results showed a nearly linear relationship between HEI-2015 and AAC score (Figure 2) after adjusting for all covariates. The trends were consistent with regressions.

### 3.3. Subgroup analysis

In the subgroup analysis (Table 4), grouped by age, gender, BMI, diabetes, hypertension, angina/angina pectoris history, smoking status, and alcohol consumption, the trends of β-coefficients were consistent and no statistically significant interaction was found in all stratification after adjusting for covariates, suggesting that the association of HEI-2015 with AAC score was robust among different population groups, and the stratification factors were not the main factors to reduce AAC score (P for interaction >0.05).

**Table 4:** Subgroup analysis.

**Stratification**	**AAC-24 score**		**AAC-8 score**	
	β **(95%CI)**	***P*** **for interaction**	β **(95%CI)**	***P*** **for interaction**
**Gender**				
Male	−0.070 (−0.197, 0.057)	0.243	−0.040 (−0.088, 0.007)	0.421
Female	−0.174 (−0.303, −0.045)		−0.067 (−0.115, −0.019)	
**Age (years)**				
< 65	−0.051 (−0.159, 0.058)	0.216	−0.029 (−0.069, 0.011)	0.268
≥65	−0.178 (−0.353, −0.002)		−0.071 (−0.137, −0.006)	
**BMI (kg/m** ^2^ **)**				
< 30	−0.140 (−0.252, −0.029)	0.462	−0.057 (−0.099, −0.016)	0.578
≥30	−0.071 (−0.227, 0.085)		−0.038 (−0.096, 0.021)	
**Serum phosphorus (mg/dL)**				
< 4	−0.141 (−0.255, −0.025)		−0.071 (−0.114, −0.028)	
≥4	−0.086 (−0.230, 0.059)	0.546	−0.023 (−0.077, 0.031)	0.156
**Serum calcium (mg/dL)**				
< 10	−0.115 (−0.212, −0.019)		−0.050 (−0.086, −0.014)	
≥10	−0.147 (−0.454, 0.160)	0.844	−0.077 (−0.192, 0.038)	0.656
**Hypertension (%)**				
Yes	−0.023 (−0.179, 0.133)	0.122	−0.013 (−0.071, 0.045)	0.090
No	−0.168 (−0.279, −0.057)		−0.073 (−0.114, −0.031)	
**Diabetes (%)**				
Yes	−0.087 (−0.307, 0.133)	0.734	−0.063 (−0.145, 0.019)	0.805
No	−0.128 (−0.229, −0.027)		−0.052 (−0.089, −0.014)	
**Angina/Angina pectoris history (%)**				
Yes	−0.188 (−0.727, 0.351)	0.804	−0.123 (−0.324, 0.078)	0.489
No	−0.119 (−0.214, −0.025)		−0.052 (−0.087, −0.017)	
**Smoking status (%)**				
Never smoking	−0.101 (−0.228, 0.026)	0.214	−0.044 (−0.091, 0.003)	0.120
Former smoker	−0.036 (−0.278, 0.206)		−0.016 (−0.106, 0.074)	
Current smoker	−0.231 (−0.409, −0.054)		−0.103 (−0.169, −0.038)	
**Alcohol consumption(%)**				
Current drinkers	−0.137 (−0.247, −0.027)	0.575	−0.057 (−0.098, −0.016)	0.931
Ex-drinkers	−0.075 (−0.358, 0.209)		−0.065 (−0.171, 0.040)	
Current smoker	−0.090 (−0.376, 0.196)		−0.033 (−0.139, 0.074)	

The subgroup analyses were adjusted for all covariates except the stratification variable itself.

## 4. Discussion

In the study, to explore the relationship between the HEI-2015 and AAC, we applied large-scale and representative data from 2013 to 2014 NHANES. The results indicated that higher HEI-2015, representing greater compliance with 2015–2020 DGA, was related to a lower AAC score and decreased risk of having AAC. Moreover, participants in the optimal group were less likely to have AAC and higher AAC score, and consuming the recommended intake of fruits, greens, and beans was associated with a lower AAC score. The subgroup analysis showed that the negative correlation of HEI-2015 with AAC was consistent with the overall results.

The relationship between dietary patterns and vascular calcification has received attention from investigators and has been explored in previous studies, although there is no consistent conclusion. A cross-sectional investigation of 5,042 subjects based on the Multi-Ethnic Study of Atherosclerosis (MESA) showed that both comprehensive healthy dietary pattern (the sum of weighted categorical levels of 36 food groups) and simplified healthy dietary pattern (whole grains, seeds and nuts, fruit, fried potatoes, processed meats, and added fats and oils) were not correlated with coronary artery calcification (CAC) (34). A prospective cohort study based on population, of data from the Heinz Nixdorf Recall (HNR) study, supported that a “Mediterranean-like” diet was correlated with a slower progression of CAC and a lower degree of CAC (35). The inconsistency of the results might be due to the different components of the dietary patterns or different population groups; therefore, more studies are needed for further exploration. Our study focused on the association of adherence to DGA with AAC, more appropriate to the national context, compared with regional dietary patterns or custom dietary patterns. Furthermore, the abdominal aorta is prone to calcification (36) and attention to its calcification can be beneficial for earlier prevention. Thus, this was the first study concentrating on the relationship of HEI-2015 with AAC.

Calcified plaques in the coronary arteries indicate a 1.7-fold increased risk of mortality (37), which has attracted widespread attention; whereas, the mechanism underlying vascular calcification is not well understood. Some studies have shown that the process of vascular calcification may be associated with or driven by inflammatory abnormalities (38), leading to atherosclerosis (39). Therefore, the inflammatory process, as an important component of vascular calcification, has been recognized and anti-inflammatory therapy may theoretically help to reduce calcification (40). The discovery of this inflammatory mechanism provides some explanatory evidence for our results.

Dietary intake regulates inflammation and is one of the potential avenues for the prevention of chronic diseases (41). Previous studies have emphasized the description of the relationship between diet and inflammation through dietary patterns. A cross-sectional study of 667 individuals from the Malmo Diet and Cancer study found that higher diet quality, indicating greater adherence to the Swedish Nutrition Recommendations, was linked to lower levels of inflammation (42). Moreover, higher HEI-2015 was proved to be correlated with inflammatory biomarkers in middle-to-older aged adults, according to data from The Cork and Kerry Diabetes and Heart Disease Study (43). Therefore, higher HEI-2015, meaning more intake of adequacy components and/or less intake of moderation components, may improve vascular calcification by reducing inflammation levels. From the component analysis of HEI-2015 in our study, it was shown that consuming the recommended intake of fruit, greens, and beans was more likely to have a lower AAC score, which was in accordance with the results of the previous study. A longitudinal cohort with 20 years of follow-up, including 2,506 young and healthy subjects with a mean age of 25.3 (SD 3.5) years, indicated that higher intake of vegetables and fruits during young adulthood was related to decreased risk of prevalent CAC (44).

From the perspective of mechanisms, vascular calcification is considered to be a chronic inflammatory process, initiated by activated macrophages (45) that secrete pro-inflammatory factors to promote differentiation from vascular smooth muscle cells (VSMCs) to bone cells (46). Among the pro-inflammatory factors, TNF-α, mainly produced by the M1 macrophages, was found to induce the expression of alkaline phosphatase (ALP) *via* the NF-κB pathway, and in turn, promote calcification in VSMCs (47). Moreover, experiments have shown that extracted components of fruits, green vegetables, and beans, such as flavonoids and soyasaponins, could reduce the release of TNF-α and nitric oxide (NO), suppress inducible NOS (iNOS) expression, an effect associated with the inhibition of the NF-κB pathway, to produce anti-inflammatory responses (48, 49). Therefore, components of HEI-2015 may play an anti-inflammatory role and prevent vascular calcification by influencing the mechanisms mentioned earlier. From both clinical and mechanistic points of view, the components of dietary patterns are associated with the inhibition of vascular calcification. Analyzing the correlation of HEI-2015 with AAC was conducive to clarifying the effect of the dietary pattern that adheres to 2015–2020 DGA on vascular calcification and its related diseases including CVD.

There are several strengths in this study. It was the first one to examine the association of HEI-2015 with AAC. Meanwhile, all the data we used were obtained from NHANES, which has a standardized process of data collection to ensure data accuracy. By using a population-based sample for analysis, the conclusions can be widely generalized to the American population. Regarding sensitivity analysis, consistent results were obtained using both AAC-24 and AAC-8 scores. Moreover, HEI-2015 and AAC were analyzed as continuous variables and as categorical variables, respectively, and an inverse relationship between HEI-2015 and AAC score and the presence of AAC were consistently observed, indicating a highly reliable conclusion. We also conducted subgroup analysis and found that there was no obvious interaction in the stratification of gender, age, BMI, hypertension, diabetes, and angina/angina pectoris history, implying that the above stratification factors could not change the inverse correlation of HEI-2015 with AAC, thus, the relationship was relatively stable.

Some limitations require to be noted. First, owing to the nature of the cross-sectional study, causality between the HEI-2015 and AAC was not able to be confirmed, emphasizing the need for a longitudinal study to clarify results. Second, data from HEI-2015 were calculated by using 24-h dietary recall, which was prone to bias. Third, short-term assessments may overlook the characteristics of dietary habits, which tend to vary over time. Although the use of HEI allows for the translation of dietary data into more stable dietary patterns (50), cohort studies with long follow-ups are warranted to confirm our results. In addition, it was inevitable that some unknown or uncollected confounding factors might affect the results, such as the information on medications that might influence vascular calcification. Finally, data for the study were derived from NHANES, which focused on populations in the US, and the dietary guidelines were designed for Americans. All participants in the study were 40 years and older due to the limitations of AAC measurement. Since the occurrence of vascular calcification is age-related (51) and the middle-aged and elderly population is more prone to vascular calcification, our study focused on the general adult population aged 40 years and older, which is of greater significance for the prevention of vascular calcification. However, whether the association is applicable in other countries or among young subjects requires further research.

## 5. Conclusion

This cross-sectional study demonstrated that HEI-2015 had an inverse association with AAC score and the risk of AAC. The results implied that higher adherence to 2015–2020 DGA, assessed by HEI-2015, might be beneficial for preventing vascular calcification, especially for obtaining optimal HEI-2015 and consuming the recommended intake of fruits, greens, and beans, which provided some relevant implications for future prospective research. A better understanding of the relationship between dietary patterns and vascular calcification is instrumental in providing dietary recommendations for preventing vascular calcification and CVD.

## Data availability statement

Publicly available datasets were analyzed in this study. This data can be found at: https://www.cdc.gov/nchs/nhanes/.

## Ethics statement

The studies involving human participants were reviewed and approved by the NCHS Ethic Review Board. The patients/participants provided their written informed consent to participate in this study.

## Author contributions

HX designed the study. JJ drafted the manuscript and analyzed the data. DM and TW collected data. ZZ made the forms. LZ graphed the pictures. JZ reviewed the manuscript. All authors contributed to the article and approved the submitted version.
