# Supplementary material for: Association between healthy eating index-2015 and abdominal aortic calcification among US Adults
Source: Front Nutr. 2023 Jan 18;9:1027136. doi: 10.3389/fnut.2022.1027136 (PMC9889545; doi:10.3389/fnut.2022.1027136)
Supplement: Supplementary file 1 [file Data_Sheet_1.docx]

Supplementary Material

Supplementary Table 1. Description of covariates.

Supplementary Table 2. Associations of 10-unit increments of HEI-2015 with AAC-24 score and the risk of AAC (alcohol consumption was used as a categorical variable for adjustment).

Supplementary Table 3. Associations between components of HEI-2015 and AAC score (alcohol consumption was used as a categorical variable for adjustment).

# Supplementary Table 1. Description of covariates.

| Variable | Section | Description |
| --- | --- | --- |
| age | Demographics Data | Age in years of the participant at the time of screening. |
| gender | Demographics Data | Gender of the participant. |
| race/ethnicity | Demographics Data | Mexican American group and Other Hispanic group are combined into the Hispanic for analysis. |
| education | Demographics Data | - |
| poverty ratio | Demographics Data | The poverty ratio was calculated by dividing family income by the 2013 to 2014 Department of Health and Human Services’ (HHS) poverty guidelines, specific to family size, as well as the appropriate year and state. |
| body mass index (BMI) | Examination Data | kg/m**2. |
| cholesterol | Laboratory Data | - |
| creatinine | Laboratory Data | - |
| serum phosphorus | Laboratory Data | - |
| serum calcium | Laboratory Data | - |
| diabetes | Questionnaire Data | Question: {Other than during pregnancy, {have you/has SP}/ {Have you/Has SP}} ever been told by a doctor or health professional that {you have/{he/she/SP} has} diabetes or sugar diabetes? |
|  | Laboratory Data | Glycohemoglobin> 6.5% |
|  | Laboratory Data | Fasting glucose≥7.0 mmol/L |
|  | Laboratory Data | random blood glucose≥11.1 mmol/L |
|  | Laboratory Data | Two Hour Glucose (OGTT)≥11.1 mmol/L |
| hypertension | Questionnaire Data | Question: {Have you/Has SP} ever been told by a doctor or other health professional that {you/s/he} had hypertension, also called high blood pressure? |
|  | Examination Data | Average SBP≥140 mmHg, average DBP≥90 mmHg |
|  |  |  |
| angina/angina pectoris history | Questionnaire Data | Question: Has a doctor or other health professional ever told {you/SP} that {you/s/he} . . .had angina (an-gi-na), also called angina pectoris? |
| smoking status | Questionnaire Data | Never smoking: smoked less than 100 cigarettes in life; Current smoker: smoked at least 100 cigarettes in life and smoke now; Former smoker: smoked at least 100 cigarettes in life, but now quit smoking; Smoker: smoked at least 100 cigarettes in life. |
| alcohol consumption | Questionnaire Data | Alcohol consumption was defined as total alcohol consumption in the past 12 months. A drink: a 12 oz. beer, a 5 oz. glass of wine, or one and half ounces of liquor.  Non-drinkers: had less than 12 alcohol drinks in lifetime; Ex-drinkers:had less than 12 alcohol drinks a year, but at least 12 alcohol drinks in lifetime; Current drinkers: had at least 12 alcohol drinks a year. |
| daily energy intake | Dietary Data | the average of two rounds of 24-hour interview recall data on energy intake. |

# Supplementary Table 2. Associations of 10-unit increments of HEI-2015 with AAC-24 score and the risk of AAC (alcohol consumption was used as a categorical variable for adjustment).

|  | β /OR (95%CI) | P value |
| --- | --- | --- |
| Risk of AAC | 0.917 (0.850, 0.989) | 0.025 |
| AAC-24 score | -0.127 (-0.221, -0.033) | 0.008 |
| Groups of HEI-2015 |  |  |
| Inadequate | Reference |  |
| Average | -0.145 (-0.415, 0.124) | 0.291 |
| Optimal | -0.489 (-0.864, -0.115) | 0.011 |
| P for trend | 0.02 |  |
| AAC-8 score | -0.055 (-0.090, -0.019) | 0.002 |
| Groups of HEI-2015 |  |  |
| Inadequate | Reference |  |
| Average | -0.064 (-0.166, 0.037) | 0.213 |
| Optimal | -0.195 (-0.336, -0.055) | 0.007 |
| P for trend | 0.01 |  |

*Age, gender, race, BMI, cholesterol, creatinine, serum phosphorus, serum calcium, hypertension, diabetes, angina/angina pectoris history, smoke status, alcohol consumption, daily energy intake, education and poverty ratio were adjusted.*

*HEI-2015, Healthy eating index-2015.*

*Abbreviation: CI, confidence intervals.*

*OR, odds ratio.*

# Supplementary Table 3. Associations between components of HEI-2015 and AAC score (alcohol consumption was used as a categorical variable for adjustment).

|  | AAC-24 | | AAC-8 | | |
| --- | --- | --- | --- | --- | --- |
|  | β (95%CI) | P value | β (95%CI) | P value |  |
| Components of HEI-2015 |  | |  | |  |
| Total Vegetables | 0.017 (-0.067, 0.101) | 0.689 | 0.001 (-0.031, 0.032) | 0.961 |  |
| Greens and Beans | -0.090 (-0.145, -0.034) | 0.002 | -0.030 (-0.051, -0.009) | 0.006 |  |
| Total Fruits | -0.095 (-0.159, -0.030) | 0.004 | -0.042 (-0.067, -0.018) | <0.001 |  |
| Whole Fruits | -0.066 (-0.124, -0.007) | 0.028 | -0.030 (-0.052, -0.008) | 0.007 |  |
| Whole Grains | -0.026 (-0.063, 0.010) | 0.157 | -0.005 (-0.018, 0.009) | 0.501 |  |
| Total Dairy | 0.007 (-0.033, 0.047) | 0.728 | 0.001 (-0.014, 0.016) | 0.908 |  |
| Total Protein Foods | -0.048 (-0.176, 0.080) | 0.461 | -0.025 (-0.072, 0.023) | 0.317 |  |
| Seafood and Plant Proteins | -0.007 (-0.065, 0.050) | 0.806 | -0.012 (-0.033, 0.010) | 0.298 |  |
| Fatty Acids | -0.019 (-0.054, 0.017) | 0.310 | -0.008 (-0.021, 0.006) | 0.265 |  |
| Refined Grains | 0.016 (-0.021, 0.052) | 0.399 | 0.002 (-0.011, 0.016) | 0.729 |  |
| Sodium | -0.038 (-0.077, -0.001) | 0.050 | -0.014 (-0.028, 0.001) | 0.055 |  |
| Added Sugars | -0.020 (-0.060, 0.022) | 0.352 | -0.009 (-0.024, 0.007) | 0.280 |  |
| Saturated Fats | -0.022 (-0.061, 0.016) | 0.256 | -0.011 (-0.025, 0.004) | 0.147 | |

*Adjusted for age, gender, race, BMI, cholesterol, creatinine, serum phosphorus, serum calcium, hypertension, diabetes, angina/angina pectoris history, smoke status, alcohol consumption, daily energy intake, education and poverty ratio.*

*Abbreviation: CI, confidence intervals.*

*HEI-2015, Healthy eating index-2015.*
